# O-51 - POST-PROCESS ENVIRONMENTAL CONTAMINATION DRIVING A PROLONGED SALMONELLA INFANTIS OUTBREAK IN THE UK

**DOI:** 10.1093/pubmed/fdag046.045

**Published:** 2026-07-09

**Authors:** Ms Elaine Forester

**Affiliations:** UK Health Security Agency

## Abstract

**Background:**

Between 2020 and 2022, a prolonged national outbreak of Salmonella enterica serovar Infantis in the United Kingdom was linked to ready to eat pork scratchings. Following detection of linked Salmonella isolates from human cases and pork scratchings through routine whole genome sequencing (WGS) surveillance, multi agency investigations focused on food production environments and manufacturing controls. Factory investigations demonstrated that cooking processes were sufficient to eliminate Salmonella from raw pork rind; however, the outbreak strain was isolated from finished product, post process equipment, drains, and floors within a single manufacturing facility. Structural and hygienic deficiencies were identified, including limited segregation between low and high care areas, inappropriate drains, damaged flooring, and cleaning practices capable of generating aerosolisation, all consistent with post process environmental contamination and persistence. Phylogenetic analysis showed minimal genetic diversity between human, food, and environmental isolates, supporting a common source with prolonged contamination. In response, production was halted, remedial engineering and hygiene controls were implemented, and large scale product recalls were initiated. A subsequent national microbiological survey of 605 pork scratching products (2022) demonstrated a low overall prevalence of Salmonella (0.3%) but identified contaminated products from a different manufacturer which instigated another factory investigation and product recall.

**Objectives:**

This presentation will focus on environmental investigations and the need for robust post cook environmental controls, appropriate hazard assessment for low water activity foods, and strengthened recall implementation to prevent prolonged foodborne outbreaks

**Methods:**

Not applicable

**Results:**

Results of the follow-up survey will be briefly discussed

**Conclusions:**

This talk will focus on factory investigations in a prolonged Salmonella outbreak. Ultimately this outbreak resulted in a guilty plea from the manufacturer and was the first prosecution in UK history to use WGS to link human cases to contaminated product

